# A flexible software tool for fitting the parameters of neuronal models

**DOI:** 10.1186/1471-2202-14-S1-P202

**Published:** 2013-07-08

**Authors:** Péter Friedrich, Attila I Gulyás, Tamás F Freund, Szabolcs Káli

**Affiliations:** 1 Institute of Experimental Medicine, Hungarian Academy of Sciences, Budapest, Hungary; 2 Faculty of Information Technology, Pázmány Péter Catholic University, Budapest, Hungary

## 

Multi-compartmental conductance-based neuronal models have the potential to imitate the behavior of real neurons with great accuracy. However, these models have many parameters, often poorly constrained by the available data. One alternative is to utilize much simpler (e.g., reduced compartmental or integrate-and-fire type) model neurons, whose non-biophysical parameters need to be set such that the behavior of the model cell best approximates that of the real neuron.

For both simple and detailed models, the relationship between the values of the parameters and the behavior of the model is nonlinear and complex, and the task of finding the optimal parameter values is highly non-trivial. Thus, a reliable automated parameter-constraining procedure which could handle the different scenarios relevant for model fitting and model simplification would be of great value, and the goal of our research was to develop such a flexible framework.

The result of our efforts is the software called Optimizer, implemented in the Python programming language. The program consists of five independent modules, which are connected via a sixth core module. These modules are responsible for reading and handling the target data, interfacing with the simulator, storing the settings specified by the user, as well as implementing the error functions and the optimization algorithms. The program can run optimizations based on one or more traces, and currently implements nine commonly used error functions based on either point-by-point comparisons or extracted features, including mean squared error, spike count, and action potential characteristics. Arbitrary linear combinations of these error functions are also supported. For optimization, the program relies on the inspyred package, which implements a wide selection of powerful nonlinear optimization algorithms; our default choice is a canonical genetic algorithm. The software can be easily modified, expanded, and customized to suit individual needs. For example, the user can implement his/her own specific error function simply by creating the function in the appropriate file and adding the name to the list of the functions. The program currently supports the Neuron simulator, but it can be easily extended to support other simulation software. Optimizer provides two different interfaces. One is a graphical interface, which allows the user to set the optimization parameters and observe, analyze the results in a convenient way. The other interface is command-line based, which reads the settings from a text file, and thus allows the optimization to run on platforms that do not support graphical interactions.

So far, we tested the program on four different types of problems: we used it to determine conductance densities in squid axon and in a multi compartmental CA1 pyramidal cell; we fitted the passive parameters of a reconstructed neuron; we fitted a nonlinear integrate-and-fire model to complex biological data; and we successfully applied the software in two different cases of systematic model simplification. In all tests, Optimizer showed equally good or better performance than software solutions described in the literature. Its simplicity and remarkable flexibility make the software applicable to a wide range of problems, making it an extremely useful research tool.

